# Supplementary material for: Acute paracoccidioidomycosis worsened by immunosuppressive therapy due to a misdiagnosis of Crohn’s disease
Source: PLoS Negl Trop Dis. 2023 Jan 10;17(1):e0011023. doi: 10.1371/journal.pntd.0011023 (PMC9870162; doi:10.1371/journal.pntd.0011023)
Supplement: Attachment — Submitted filename: Acute Paracoccidioidomycosis Paper edits.docx [file pntd.0011023.s001.docx]

Acute Paracoccidioidomycosis worsened by immunosuppressive therapy due to a misdiagnosis of Crohn’s Disease

Edits by section

**Abstract**

- “Recently, modifications in the profile of the disease have been described in Rio de Janeiro, Brazil”—I am not clear what this means, does not make sense to me. Needs clarification.
- In the 4^th^ sentence, Crohn’s Disease is misspelled as “Chron disease”. Please be consistent.
- “After seven months of treatment, the patient presented [with] acute abdominal pain which led to an emergent appendectomy…”
- “After 24 months, the patient reached…”
- Consider revising the last sentence phrasing: “… the risks of initiating immunosuppressive therapy before excluding fungal infections…”

**Presentation of Case**

- Line 26: remove the word “patient”
- Line 27: “presenting with ulcerated lesions…”
- Line 28: instead of “He referred…” considering “He had chronic diarrhea and abdominal pain for the past 3 years…”
- Line 29: Crohn’s Disease—please be consistent.
- Line 29-30: consider removing “and computed tomography”. While I am not a gastroenterologist, I’m not sure a CT scan is used for the diagnosis of Crohn’s Disease. I’m not quite sure a CT scan is the modality of choice to reveal erosions and a vegetative lesion as well.
- Line 32-33: “in a few months, with evolving odynophagia, a 20 kg weight-loss” There should not be a “-“ between kg and weight.
- Line 41-42: Consider revision of the sentence to the following: “Since the patient was clinically stable and refused hospitalization, we decided to start…”. You mention that the patient had a sulfa allergy, I’m not sure why that is necessary to say. May need to clarify if you want to keep that part of the sentence in. Is that a contraindication to certain treatments for Paracoccidioides?
- Line 43: should clarify that you used oral itraconazole
- Line 43: consider revision: “…to ensure optimal absorption.”
- Line 44: consider revision: “…was prescribed for suspected Paracoccidioides-related adrenal insufficiency”.
- Line 45: “evolved with progressive healing”—this is poor phrasing, please reword
- Line 46-47: “presented with acute abdominal pain, which led to an emergent appendectomy.” The word “surgery” is not necessary at the end.
- Line 48: Consider revision: “Treatment continued for 24…”
- Line 50-51: Consider revision: “…negative serologic DID titers, and no evidence of disease on CT imaging and a repeat Colonoscopy revealed healing lesions without evidence of fungal organisms on histopathologic examination.”
- Line 51-52: Should directly state if the GI symptoms completely resolved. Should directly state if cutaneous lesions resolved. And if you have an after picture of the patient’s nose to show that the lesions are well healed, that would be great. Did the patient regain his weight that he lost?
- Line 52: it’s not really discontinuing treatment… perhaps saying something like “off treatment for 2 years”
- General comments:
  - Should state somewhere the patient’s HIV status even if negative
  - Should mention patient’s comorbidities (or lack of)
  - Did the Paracoccidioides grow in culture? If yes, any susceptibilities and MICs obtained?
  - What guided duration of treatment with Itraconazole? Why 24 months? What did you monitor to determine that the patient should be on 24 months vs 18 months vs 36 months, etc?

**Case Discussion**

- In general, well written.
- Line 69: perhaps it is the “disruption” of the soil rather than the “management” of the soil? Consider the wording
- Line 77-78: I’m not entirely clear on what the Peyer’s patches have to do with the underdiagnosis of GI related Paracoccidioides infection. Does Paracoccidioides have a pre-disposition of the Peyer’s patches? Is that usually where fungal elements are found?
- Line 103: should say “oral itraconazole”
- Line 104: consider revision: “he did not have any further complications.”
- General comments:
  - Consider mentioning how the Crohn’s Disease misdiagnosis phenomena has also been documented with Histoplasmosis—this may also add to your key learning point #2.

**Key learning points**

- The first key learning point is supported in the article.
- The second key learning point should be reworded. The phrase "before excluding fungal infections" almost implies that fungal infections are routinely screened prior to initiation of immunosuppressive agents. Consider something along the line of... "The risk of fungal infections (or reactivations) should be considered prior to initiating immunosuppressive therapies."
- The third key learning point did not come through in the article. It was not clear to me where the multidisciplinary aspect was discussed.
