# Supplementary material for: Acute paracoccidioidomycosis worsened by immunosuppressive therapy due to a misdiagnosis of Crohn’s disease
Source: PLoS Negl Trop Dis. 2023 Jan 10;17(1):e0011023. doi: 10.1371/journal.pntd.0011023 (PMC9870162; doi:10.1371/journal.pntd.0011023)
Supplement: Attachment — Submitted filename: rebuttalletter.docx [file pntd.0011023.s002.docx]

Evandro Chagas National Institute of Infectious Diseases, INI/Fiocruz

Avenida Brasil, 4365, Manguinhos

CEP 21040-900 - Rio de Janeiro - RJ - BRAZIL

Phone: +55 (21) 38659578

E-mail: eduardo.falcao@ini.fiocruz.br

November 30^th^, 2022

Dear Academic Editor and Reviewers,

We sincerely appreciate the thoughtful review provided for our manuscript “Acute Paracoccidioidomycosis worsened by immunosuppressive therapy due to a misdiagnosis of Crohn’s Disease” (PNTD-D-22-01328). Addressing the constructive comments has improved our article. All suggestions were considered and we managed to answer and solve all the possible issues. Our point-by-point responses to the Reviewers’ comments can be found below. Please consider the file with tracked changes to see the lines indicated in the answers.

Reviewer #1:

1- The first key learning point is supported in the article.

The second key learning point should be reworded. The phrase "before excluding fungal infections" almost implies that fungal infections are routinely screened prior to initiation of immunosuppressive agents. Consider something along the line of... "The risk of fungal infections (or reactivations) should be considered prior to initiating immunosuppressive therapies."

The third key learning point did not come through in the article. It was not clear to me where the multidisciplinary aspect was discussed.

**Thank you for the suggestion. We made some changes and included a phrase to discuss the multidisciplinary care. Please see lines 132-134 and 141-148.**

2- Needs revisions, though largely minor, would not accept as is.

**Indeed. We appreciate and accept all the suggestions sent in the attached file. Please see lines 26-60.**

3- The case description had a lot of grammatical errors that sometimes made it confusing or difficult to read through smoothly. The case discussion, however, was well written. I thought that this was important to point out as describing the case is almost equally as important as your discussion because other readers may be referring to the authors' case to compare their own patient. Moreover, it may deter readers from actually reading the article if it does not flow smoothly.

**We agree. We made several changes in the case description section.**

4- Should state somewhere the patient’s HIV status even if negative. Should mention patient’s comorbidities (or lack of)

**Thank you for this contribution. Please see lines 26 and 43.**

5- Did the Paracoccidioides grow in culture? If yes, any susceptibilities and MICs obtained?

**Thank you for your comment. Actually, we forgot to include this important information. We included a sentence about the fungal isolation in culture (lines 40 and 41). Concerning antifungal susceptibility tests, they are not routinely performed for the clinical assistance of patients with PCM and are not recommended by the Brazilian Consensus on PCM. These tests are hard to perform for filamentous and fastidious fungi and previous studies have shown that *Paracoccidioides* spp. are highly susceptible to antifungal drugs.**

6- What guided duration of treatment with Itraconazole? Why 24 months? What did you monitor to determine that the patient should be on 24 months vs 18 months vs 36 months, etc?

**According to the Brazilian Guidelines for the clinical management of PCM, the time of treatment with itraconazole ranges from 9 to 18 months, but each case should be specifically evaluated, some patients need more time to reach cure criteria. In the case we reported, the patient was treated until clinical and serological cure were achieved, as described in lines 53 to 58.**

5- Line 69: perhaps it is the “disruption” of the soil rather than the “management” of the soil? Consider the wording

**We understand the Reviewer’s point. However, soil management involves several activities, mainly agricultural and not all those infected with PCM worked directly with soil disruption**

6- Line 77-78: I’m not entirely clear on what the Peyer’s patches have to do with the underdiagnosis of GI related Paracoccidioides infection. Does Paracoccidioides have a pre-disposition of the Peyer’s patches? Is that usually where fungal elements are found?

**As we described in line 82, the acute form of PCM traditionally affects the reticuloendothelial system, notably lymph abdominal organs, including the aggregated lymphoid nodules of the Peyer's patches, which can progress to GI disease. As the symptoms of GI involvement of PCM are unspecific and endoscopic examination of the GI tract is invasive and not routinely recommended, the underdiagnosis of this clinical presentation is expected. Please see lines 83-86.**

7- Consider mentioning how the Crohn’s Disease misdiagnosis phenomena has also been documented with Histoplasmosis—this may also add to your key learning point #2.
**Thank you for this suggestion. Please see lines 135-138.**

Reviewer #2

1. Line 30: The authors mention that treatment with mesalazine and prednisone in immunosuppressive doses was prescribed. The authors should consider including the doses.

**Thank you for this contribution. Please see line 31.**

2. Line 79: The authors cite a review of 46 cases of gastrointestinal PCM. Does the presentation of the current case, e.g. chronicity of symptoms or cutaneous lesions after starting immunosuppressive therapy, differ from the previously reported cases? The authors might consider briefly contrasting this case with a previously reported case of gastrointestinal PCM.

**Thank you. We agree. Please see lines 87-89.**

3. Line 99: The authors mention that IV antifungals are usually indicated in gastrointestinal involvement as per Brazilian guidelines which was not possible in this case as the patient declined hospitalization. The authors could consider including the IV antifungals of choice, e.g. IV liposomal amphotericin B for 2-4 weeks. PMID: 28746570

**Thank you for this contribution. Please see lines 109-110.**

4-5. Line 107: The possible correlation between the patient’s residence near a highway and the increased incidence of PCM with highway construction is an interesting observation. The authors could consider mentioning the increase in PCM from 1.29 cases to 8.25 cases/1 million persons/y during a highway construction as per cited source as well as hypotheses of increased incidence due to clearing of forests and during times of increased soil humidity. PMID: 29048286.

Does this potential higher risk of PCM due to residence near newly constructed highways lead to health care disparities, e.g. are people with lower income more likely to live in these areas?

**Indeed. Please see lines 119-125.**

Yours sincerely,

**Eduardo Mastrangelo Marinho Falcão - MD, MSc**
